# Enhancing cognitive control in amnestic mild cognitive impairment via at-home non-invasive neuromodulation in a randomized trial

**DOI:** 10.1038/s41598-023-34582-1

**Published:** 2023-05-08

**Authors:** Kevin T. Jones, Avery E. Ostrand, Adam Gazzaley, Theodore P. Zanto

**Affiliations:** 1 Department of Neurology, University of California-San Francisco, 675 Nelson Rising Ln, San Francisco, CA 94158 USA; 2 Neuroscape, University of California-San Francisco, 675 Nelson Rising Ln, San Francisco, CA 94158 USA; 3 Departments of Physiology and Psychiatry, University of California-San Francisco, 675 18th St, San Francisco, CA 94143 USA; 4 Sandler Neurosciences Center, 675 Nelson Rising Lane, San Francisco, CA 94158 USA

**Keywords:** Cognitive ageing, Cognitive neuroscience, Learning and memory, Neural circuits, Human behaviour

## Abstract

Individuals with multi-domain amnestic mild cognitive impairment (md-aMCI) have an elevated risk of dementia and need interventions that may retain or remediate cognitive function. In a feasibility pilot study, 30 older adults aged 60–80 years with md-aMCI were randomized to 8 sessions of transcranial alternating current stimulation (tACS) with simultaneous cognitive control training (CCT). The intervention took place within the participant’s home without direct researcher assistance. Half of the participants received prefrontal theta tACS during CCT and the other half received control tACS. We observed high tolerability and adherence for at-home tACS + CCT. Within 1-week, only those who received theta tACS exhibited improved attentional abilities. Neuromodulation is feasible for in-home settings, which can be conducted by the patient, thereby enabling treatment in difficult to reach populations. TACS with CCT may facilitate cognitive control abilities in md-aMCI, but research in a larger population is needed to validate efficacy.

## Introduction

The adult aging population is rapidly growing as the Baby Boomer generation now totals over 71 million adults in the United States alone. This rise in the population of seniors is also associated with increased incidence of cognitive decline and dementias^1^, which will cause a significant burden on society unless the onset of dementia is delayed or prevented^2^. Interventions that can prolong independence, even for a single year, are estimated to save trillions of dollars per year and would be a greater economic boon than eradicating an individual disease^3^. Amnestic mild cognitive impairment (aMCI) is characterized by a decline in episodic memory^4^, whereas multi-domain aMCI (md-aMCI) is a sub-type that includes deficits in other cognitive domains, such as executive or attentional control^5^. Not only does md-aMCI adversely affect quality of life, but it poses the highest risk for progression to Alzheimer’s disease (AD)^6^. Estimates suggest 7.2% (range: 0.5–31.9%) of adults over the age of 65 have md-aMCI^7^ with a 37% chance of progressing to dementia within four years^8^. By 2050, the population of adults over 65 is expected to double and those with AD is expected to quadruple^9^. Given this public health crisis, any fruitful interventions that lead to prophylaxis of cognitive decline and prolong independence will result in a significant benefit to individuals, families, and society as a whole.

As individuals age, declines in cognitive control (working memory and attention) become noticeable and during progression to aMCI these deficits become detrimental to independent daily living^10^. The magnitude of decline in cognitive control also serves as a predictor of progression to AD, more than declines in long-term memory alone^11^. This could help explain why md-aMCI carries the highest risk for developing AD. In clinical populations with cognitive control deficits, pharmaceutical interventions remain the most common approach for managing symptoms^12^, despite limited evidence of success at improving cognitive control functioning^13^. However, recent efforts indicate preventative approaches may help delay or prevent the onset of symptoms of AD. One such approach is through cognitive control training (CCT), which has been shown to change neural circuit information processing^14^ and can persist for months^15^ or even years in healthy older adult populations^16^.

One promising technique at enhancing benefits from CCT to improve cognitive function is noninvasive transcranial alternating current stimulation (tACS), which has demonstrated evidence in enhancing cognition in healthy individuals^17,18^ as well as those with ADHD^19^, schizophrenia^20^, and stroke^21^. TACS modulates cognitive functioning through a combination of neural entrainment and resonance. The applied oscillatory electrical stimulation results in the recruitment of neurons to join a local oscillating network, that affects both local and broad network computations^22,23^. The oscillatory entrainment from tACS leads to changes in observed amplitude^24^, frequency^25^, coherence^24,26,27^, connectivity^28,29^, and cross-frequency activity^29,30^ in the EEG and in resting-state fMRI^31^ (further reviewed in:^32,33^), believed to be due to enhanced cortical plasticity^34,35^. This lasting aftereffect or plasticity can impact behavior in cognitive tasks^24,35^, making tACS an ideal tool to pair with CCT in vulnerable populations. Importantly, tACS has the advantages of being safe, painless, well-tolerated by participants and significantly cheaper than other clinical intervention techniques^36^.

Frontal theta activity, and its corresponding cognitive control functions, decline in advanced age^14^ and is even more deficient in older adults with MCI^37^. We recently demonstrated that tACS in the theta range (6 Hz) improves cognitive control in healthy younger adults across a single session of CCT^17,18^. In healthy older adults we also observed that 6 Hz theta tACS applied during CCT enhanced multitasking more than sham^38^ or 1 Hz delta^28^ tACS. These prior results guided our pre-registered approach in the current study, as we included a frequency control (delta) as opposed to a duration control (sham) in the current study. This study was designed to not only extend these results to at-home research and clinical populations, but also replicate the frequency specificity of these effects. Taken together, the goal of this study was to apply theta tACS over prefrontal cortex to assess whether facilitating theta oscillations during CCT in older adults with md-aMCI may also improve cognitive control abilities.

In the current study, we hypothesized that 5 days of theta tACS + CCT would yield larger improvements in cognitive control performance compared to control tACS + CCT, a pattern we observed in healthy older adults^38^. Moreover, we assessed whether 3 weekly “maintenance” sessions would yield additive benefits after a month (8 total sessions) of tACS + CCT. Cognitive control was assessed via performance on (1) the multitasking CCT task, (2) a sustained attention task (near transfer), and (3) a working memory task (far transfer). These three cognitive control abilities were assessed because they have each been associated with frontal theta activity^14,17,18,39^ and are affected in healthy aging and MCI populations^14,40^. To measure improvements outside of our preregistered outcomes, we included four exploratory tasks that probe various attentional control demands. If this approach was ever to be considered a viable intervention, it should be tested in real-world settings to better understand effectiveness. Indeed, we have recently demonstrated feasibility of at-home tACS, without direct researcher intervention, in a healthy young adult population^39^. Here, we aimed to extend this research to an md-aMCI population, such that tACS + CCT was conducted within the participant’s home without direct researcher assistance.

## Results

### Tolerability and adherence

To measure adherence to the remote study schedule, we assessed the percentage of training and outcome sessions completed. Comparing between groups revealed no significant difference on the training adherence (Theta: 96.43% (SD: 0.08), Control: 97.12% (SD: 0.10); t = 0.20, p = 0.84, Cohen’s d = 0.08) or outcome measures (Theta: 91.67% (SD: 0.11), Control: 89.74 (SD: 0.16); t = -0.37 p = 0.72, Cohen’s d = 0.14). Notably, the tACS side effects reported were mild for all 11 categories (scale 0–10), and there was no significant difference between the groups for all 11 side effect metrics (Table 1), even when uncorrected for multiple comparisons.

**Table 1:** Average side effects across all eight tACS sessions for the theta and control groups.

Side effect	Theta average (SD)	Control average (SD)	t value	*p* value	Cohen’s d
Headache	0.11 (0.24)	0.04 (0.09)	0.88	0.39	0.35
Neck pain	0.23 (0.36)	0.03 (0.09)	1.85	0.078	0.74
Scalp pain	0.04 (0.10)	0.03 (0.06)	0.42	0.675	0.17
Tingling	0.91 (0.73)	0.86 (1.47)	0.11	0.913	0.04
Itching	0.12 (0.23)	0.07 (0.22)	0.64	0.53	0.26
Burning sensation	0.22 (0.49)	0.77 (1.06)	1.70	0.103	0.68
Increased alertness	0.82 (1.62)	0.23 (0.44)	1.15	0.260	0.47
Increased sleepiness	0.30 (0.70)	0.07 (0.17)	1.07	0.296	0.43
Trouble concentrating	0.27 (0.46)	0.29 (0.74)	0.09	0.933	0.03
Acute mood change	0.15 (0.34)	0.32 (0.85)	0.66	0.518	0.26
Phosphenes	0.30 (0.47)	0.03 (0.09)	1.94	0.064	0.78

Values in parentheses represent standard deviation of the mean. The *p* values are from independent samples t-tests.

### Multitasking performance

To assess alterations in multitasking performance, we first analyzed perceptual discrimination during multitasking via an rm-ANOVA with three time points (baseline, 1-week, 1-month) and the between-subjects factor of tACS group (theta, control). Results revealed a significant main effect of time (F_1.80,32.35_ = 9.36, *p* = 0.002, $\upeta _{{\text{p}}}^{{2}}$ = 0.34) such that performance improved post intervention. No main effect of group (F_1,18_ = 0.40, *p* = 0.535, $\upeta _{{\text{p}}}^{{2}}$ = 0.02) or a time × group interaction (F_1.80,32.35_ = 0.09, *p* = 0.87, $\upeta _{{\text{p}}}^{{2}}$ = 0.01) was observed. Next, we assessed alterations in visuomotor tracking via an rm-ANOVA with three time points (baseline, 1-week, 1-month) and the between-subjects factor of tACS group (theta, control). Results revealed a significant main effect of time (F_1.80,32.35_ = 15.88, *p* < 0.001, $\upeta _{{\text{p}}}^{{2}}$ = 0.47), but no time × group interaction (F_1.80,32.35_ = 1.63, *p* = 0.214, $\upeta _{{\text{p}}}^{{2}}$ = 0.06) was observed. There was a trend towards a significant between-subjects group effect (F_1.80,32.35_ = 4.32, *p* = 0.052, $\upeta _{{\text{p}}}^{{2}}$ = 0.19). Together, this pattern of results revealed that while both groups exhibited improved multitasking abilities following multitasking CCT, there was not a significant additive effect of tACS (Table 2).

**Table 2:** Performance on each outcome measure at the baseline, 1-week, and 1-month follow ups.

Cognitive domain	Metric	Theta tACS			Control tACS		
		Baseline	1-week	1-month	Baseline	1-week	1-month
MT: Perceptual Discrimination	Difficulty threshold	8.01 (1.56)	9.29 (2.83)	10.10 (1.40)	8.51 (1.65)	9.31 (2.40)	10.67 (1.18)
MT: Visuomotor Tracking	Difficulty threshold	8.57 (2.06)	10.18 (2.91)	11.54 (2.82)	10.56 (2.98)	13.61 (4.17)	13.66 (4.32)
Sustained Attention	Reaction time	526.87 (107.59)	**436.43 (73.36)**	**427.91 (63.53)**	431.95 (55.78)	416.48 (59.51)	395.48 (48.95)
Working Memory	# correctly recalled	6.13 (0.96)	6.09 (1.29)	6.13 (1.15)	6.56 (0.81)	6.89 (0.78)	6.50 (0.75)
Inhibitory Control	RCS	1.12 (0.34)	**1.32 (0.38)**	**1.42 (0.34)**	1.38 (0.16)	1.45 (0.17)	1.52 (0.17)
Attentional Orienting	RCS	2.00 (0.50)	**2.37 (0.55)**	**2.43 (0.54)**	2.36 (0.39)	2.53 (0.33)	2.67 (0.19)
Visuospatial Search	RCS	0.76 (0.16)	0.88 (0.18)	0.89 (0.19)	0.93 (0.15)	1.01 (0.14)	1.19 (0.44)

Multitasking (MT) values represent the difficulty threshold achieved during Monitor. Sustained Attention are the composite RT score for both sustained attention modules in ACE. Working Memory is the composite span score on the forwards and backwards spatial span tasks. The bottom three rows display RCS values on exploratory analyses on ACE-X tasks. Values in parentheses below the means represent standard deviation. Values in bold font indicate a significant change from baseline in a group × time interaction (p_bonf_ < 0.05).

### Sustained attention

**Figure 1:**
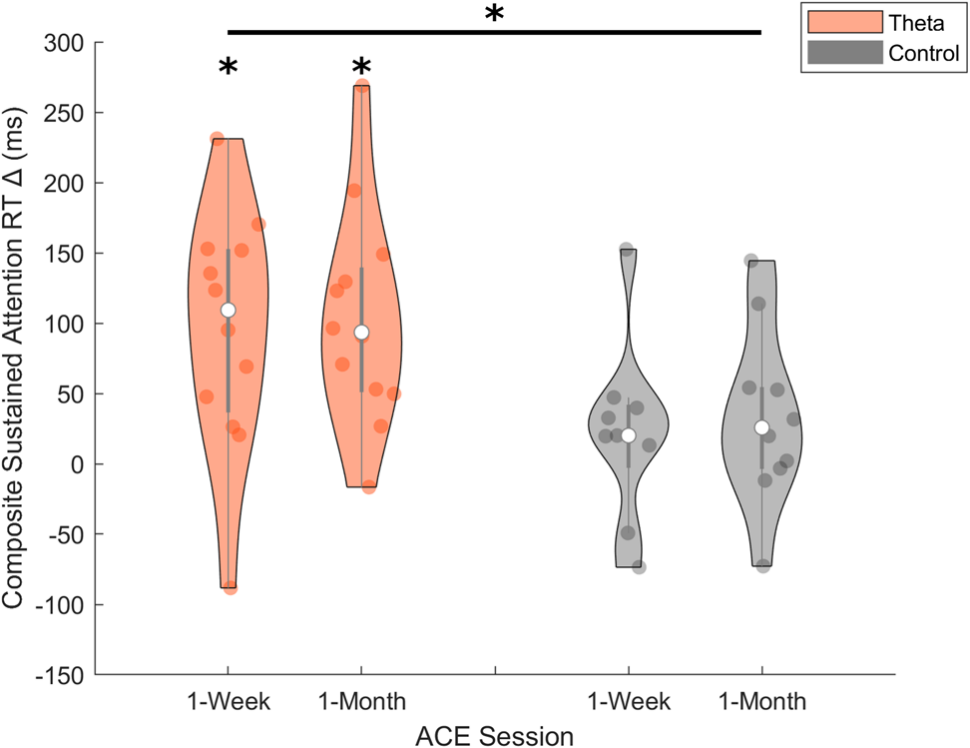
Violin plots of composite sustained attention RT data for change from baseline at 1-week and 1-month time points for theta (orange) and control (gray) tACS groups. White dots represent the median data point. *Note* values represent pre-post RT so that gains following training are higher on the y-axis. Gray bar represents the interquartile range. Colored dots (orange/gray) represent individual data points. *represents *p* of < 0.05.

To assess alterations in sustained attention we conducted an rm-ANOVA on the composite RT on the two sustained attention tasks with three time points (baseline, 1-week, 1-month) and the between-subjects factor of tACS group (theta, control). The results revealed a main effect of time (F_1.31,23.56_ = 17.34, *p* < 0.001, $\upeta _{{\text{p}}}^{{2}}$ = 0.49), such that performance improved (reduced RT) following a week of tACS + CCT (Table 2). There was no between-subjects effect of group (F_1,18_ = 2.50, *p* = 0.131, $\upeta _{{\text{p}}}^{{2}}$ = 0.12). Importantly, there was a significant time × group interaction (F_1.31,23.56_ = 6.25, *p* = 0.014, $\upeta _{{\text{p}}}^{{2}}$ = 0.49). Post-hoc comparisons revealed that this was driven by a significant difference within the theta group between baseline and 1-week time points (t = 6.16, p_bonf_ < 0.001), and baseline and 1-month time points (t = 5.95, p_bonf_ < 0.001). The control tACS group did not differ between baseline and either the 1-week (t = 1.13, p_bonf_ = 1.00) or 1-month (t = 1.66, p_bonf_ = 1.00) time points. There was no significant post-hoc effect between the 1-week and 1-month time points for the theta and control tACS groups (range of t = 0.21–0.54, all p_bonf_ = 1.00), revealing that additional tACS + CCT sessions yielded no additional benefits. Finally, no significant difference between the groups was observed at baseline (t = 3.03, p_bonf_ = 0.078). An independent samples t-test between groups on the change in RT from baseline demonstrated significantly greater gains for in the theta tACS group as compared to the control tACS group at both the 1-week (t = 2.13, *p* = 0.047, Cohen d = 0.94) and 1-month (t = 2.29, *p* = 0.033, Cohen d = 0.98) timepoints (Fig. 1). This pattern of results reveals that theta, not control tACS, enhanced sustained attention capabilities, but additional weekly booster sessions did not further enhance the performance gains beyond 1-week.

### Working memory

To assess alterations in WM capacity, we conducted an rm-ANOVA on the composite WM span score generated from the forwards and backwards span tasks with three time points (baseline, 1-week, 1-month) and the between-subjects factor of tACS group (theta, control). The results revealed no main effect of time (F_1.94,36.85_ = 0.69, *p* = 0.504, $\upeta _{{\text{p}}}^{{2}}$ = 0.04), time × group interaction (F_1.94,36.85_ = 1.09, *p* = 0.346, $\upeta _{{\text{p}}}^{{2}}$ = 0.05), or between-subjects effect of group (F_1,18_ = 1.77, *p* = 0.199, $\upeta _{{\text{p}}}^{{2}}$ = 0.09; Table 2). This pattern of results reveals that CCT with theta tACS did not affect working memory performance more than control tACS.

### Instrumental activities of daily living (IADL)

The IADL^41^ was self-administered by participants on the initial baseline session, 1-week follow-up, and 1-month follow-up. The participants overwhelmingly maxed out the rating scale (max 4) for each of the 16 categories (99.7% scores a 4 at baseline), thus making statistical analyses unnecessary (Baseline Theta tACS mean: 3.92 (SD: 0.29), Baseline Control tACS mean: 4 (SD: 0), 1-week 6 Theta tACS mean: 3.92 (SD: 0.29), 1-week Control tACS mean: 4 (SD: 0), 1-month Theta tACS mean: 4 (SD: 0), 1-month Control tACS mean: 4 (SD: 0).

### Exploratory results

**Figure 2:**
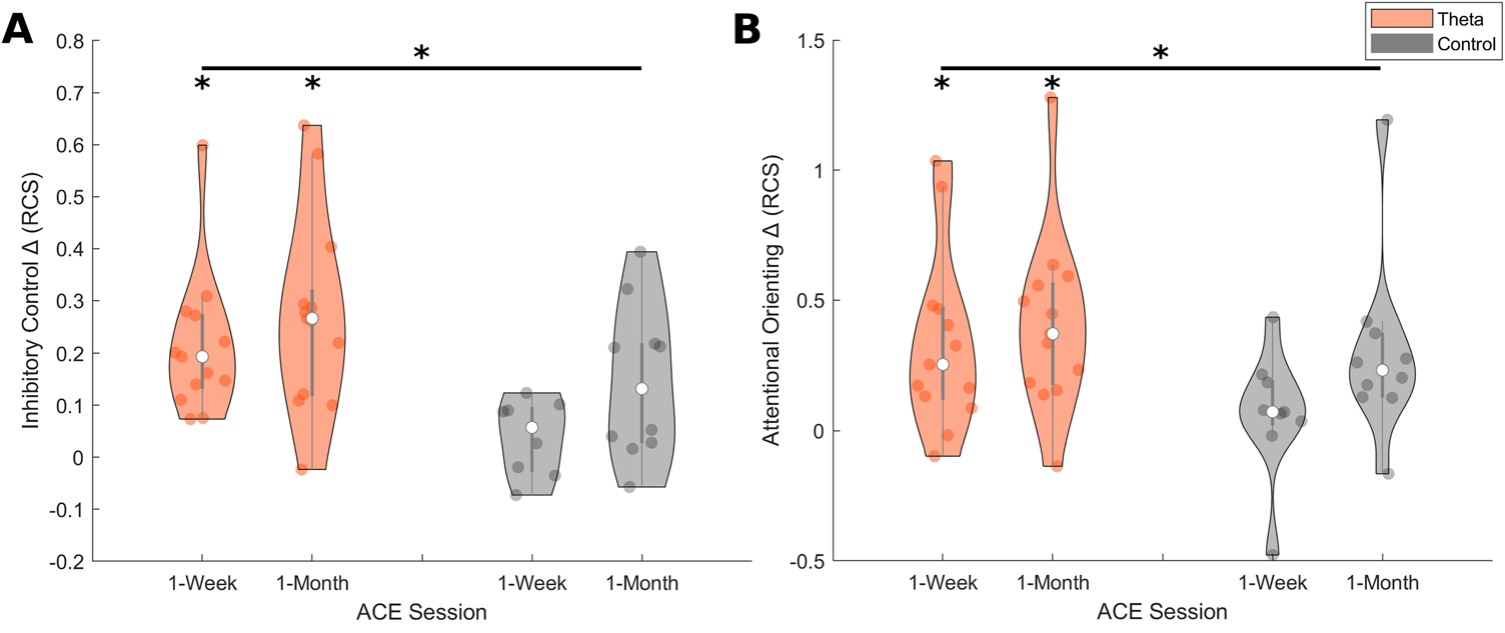
Violin plots on the Inhibitory Control (**A**) and Attentional Orienting (**B**) change from baseline rate correct score (RCS) data at 1-week and 1-month time points for theta (orange) and control (gray) tACS groups. White dots represent the median data point. Gray bar represents the interquartile range. Colored dots (orange/gray) represent individual data points. *represents *p* of < 0.05.

To assess inhibitory control, a composite rate correct score (RCS; correct trials per minute) from the Flanker and Color Tricker (Stroop) tasks were averaged together and submitted to an rm-ANOVA with three time points (baseline, 1-week, 1-month) and the between-subjects factor of tACS group (theta, control). The results revealed a main effect of time (F_1.66,29.91_ = 18.11, *p* < 0.001, $\upeta _{{\text{p}}}^{{2}}$ = 0.50; Table 2) and time × group interaction (F_1.66,29.91_ = 3.49, *p* = 0.05, $\upeta _{{\text{p}}}^{{2}}$ = 0.16), however no between-subjects effect of group (F_1,18_ = 1.63, *p* = 0.218, $\upeta _{{\text{p}}}^{{2}}$ = 0.08). Post-hoc comparisons revealed that this interaction was driven by a significant difference in the theta group between baseline and 1-week time points (t = 4.90, p_bonf_ < 0.001), and baseline and 1-month time points (t = 6.16, p_bonf_ < 0.001). The control tACS group did not differ between baseline and either the 1-week (t = 0.69, p_bonf_ = 1.00) or 1-month (t = 2.68, p_bonf_ = 0.167) follow-ups. There was no significant post-hoc effect between the 1-week and 1-month time points for the theta and control tACS groups (range of t = 1.26–1.99, range of p = 0.819–1.00), revealing that additional tACS + CCT yielded no additional benefits. Finally, no significant difference between the groups was observed at baseline (t = 1.99, p_bonf_ = 0.895). An independent samples t-test between groups on the change in RCS from baseline demonstrated no significant gains for in the theta tACS group at the 1-week (t = 0.81, p = 0.429, Cohen d = 0.36) or 1-month (t = 1.78, p = 0.09, Cohen d = 0.75) timepoints (Fig. 2A). This pattern of results reveals that theta, not control tACS, significantly enhanced inhibitory control from baseline after one week, but that these gains were not significantly different between groups. Additional weekly booster sessions did not further enhance performance gains beyond one week (Table 2).

To assess attentional orienting, RCS data from the Compass (Posner) task was submitted to an rm-ANOVA with three time points (baseline, 1-week, 1-month) and the between-subjects factor of tACS group (theta, control). The results revealed a main effect of time (F_1.89,35.87_ = 14.04, *p* < 0.001, $\upeta _{{\text{p}}}^{{2}}$ = 0.43; Table 2) and time x group interaction (F_1.89,35.87_ = 3.40, *p* = 0.047, $\upeta _{{\text{p}}}^{{2}}$ = 0.15; Fig. 2B), however no between-subjects effect of group (F_1,19_ = 2.60, *p* = 0.124, $\upeta _{{\text{p}}}^{{2}}$ = 0.12). Post-hoc comparisons revealed that the interaction was driven by a significant difference in the theta group between baseline and 1-week time points (t = 4.67, p_bonf_ < 0.001), and baseline and 1-month time points (t = 5.37, p_bonf_ < 0.001). The control tACS group did not differ between baseline and either the 1-week (t = 0.72, p_bonf_ = 1.00) or 1-month (t = 2.19, p_bonf_ = 0.523) follow-ups. There was no significant post-hoc effect between the 1-week and 1-month time points for the theta and control tACS groups (range of t = 0.70–1.47, both p = 1.00), revealing that additional tACS + CCT yielded no additional benefits. Finally, no significant difference between the groups was observed at baseline (t = 2.42, p_bonf_ = 0.346). An independent samples t-test between groups on the change in RCS from baseline approached significance, such that greater gains were observed for in the theta tACS group at the 1-week (t = 2.04, *p* = 0.055, Cohen d = 0.88) but not 1-month (t = 0.74, *p* = 0.468, Cohen d = 0.31) timepoint (Fig. 2B). This pattern of results reveals that theta, not control tACS significantly enhanced attentional orienting from baseline, but additional weekly booster sessions did not further enhance performance gains beyond one week (Table 2).

Finally, to assess visuospatial search, RCS data from the Boxed task was submitted to an rm-ANOVA with three time points (baseline, 1-week, 1-month) and the between-subjects factor of tACS group (theta, control). The results revealed a main effect of time (F_1.10,20.83_ = 5.28, *p* = 0.029, $\upeta _{{\text{p}}}^{{2}}$ = 0.22; Table 2), but no time x group interaction (F_1.10,20.83_ = 1.11, *p* = 0.311, $\upeta _{{\text{p}}}^{{2}}$ = 0.06). There was a significant between-subjects group effect (F_1,19_ = 7.88, *p* = 0.011, $\upeta _{{\text{p}}}^{{2}}$ = 0.29; Table 2).

## Discussion

This study demonstrated the feasibility of conducting remote tACS + CCT in an at-home setting with clinical populations without direct researcher supervision. The md-aMCI participants reported zero serious adverse events, overall subtle side effects, and only a 10% attrition rate (3 of 30). While we observed that each tACS group improved on the multitasking CCT task, only the theta tACS group exhibited a significant transfer of benefit to improved sustained attention ability, inhibitory control, and attentional orienting, suggesting multiple aspects of attentional control may have been facilitated by the intervention. These gains were limited to attentional tasks as visual search performance and WM capacity were not affected. Together, these results show that tACS + CCT at home is a safe and feasible means to target cognitive control in md-aMCI populations, but the beneficial effects of tACS appear limited to cognitive functions which share neural mechanisms with the CCT paradigm.

The observed 10% attrition rate is the same rate as our previous tACS studies^17,18,38,39^ as well as our previous CCT studies where no stimulation was applied^14^, all of which occurred in laboratory settings. As such, it is unlikely that neuromodulation played a role in the attrition rate. Indeed, of the three participants who did not complete the study, none reported discomfort or difficulties with neuromodulation. Demonstrating the feasibility of tACS to supplement CCT in home settings is particularly valuable during global pandemics, but it also addresses concerns regarding the inaccessibility of research environments to those with clinical disorders with limited independence in mobility or transportation.

We showed that multitasking performance was similarly improved in both the theta and delta (control) tACS groups after 5 tACS sessions, without additional gains after 8 sessions. We speculate that theta tACS may help facilitate learning the CCT task at a faster rate (i.e., within a few days), but that in time, performance in the control group will catch up to the theta tACS group due to practice effects. This interpretation is in line with our previous research demonstrating that theta tACS facilitates multitasking performance in young adults after 1 day^17,18^, but no added benefit after 5 days^39^ of tACS + CCT.

Here we observed that theta tACS + CCT exhibited a transfer of benefit to domains that share similar task demands with the divided attention CCT: sustained attention, inhibitory control, and attentional orienting (Fig. 2). Given that sustained attention has been linked to frontal theta activity^42^, applying prefrontal theta tACS likely enhanced shared neural mechanisms engaged during the multitasking training and attentional transfer tasks. Importantly, these results indicate that cognitively complex CCT tasks such as multitasking, when coupled with neuromodulation, may be used to rapidly facilitate multiple aspects of attentional control. The lack of any effect on WM capacity demonstrates that only near transfer was observed (i.e., limited to similar attention tasks). This lack of improvement on WM tasks is likely due to the CCT task not placing appreciable demands on WM networks, as the training tasks stressed the participants cognitive control skills.

In the current study we note that the three additional weekly “booster” sessions did not yield any additional benefit, although gains were maintained throughout the length of the study. Therefore, this provides guidance regarding tACS dosing, such that five (or possibly fewer) sessions is needed to improve sustained attention in an md-aMCI population. Additional research will be required to assess how long such effects may last, the proper number of sessions per individual, and if training gains also slowed future decline in related cognitive domains. Our previous research in healthy older adults showed tACS-related improvements in multitasking ability lasted at least one month^38^ and we recently observed that effects from our multitasking CCT task can sustain for years^16,43^. Thus, supplementing CCT with neuromodulation holds the potential to help alter the trajectory of cognitive decline.

Once tACS benefits become apparent, it is unclear whether additional tACS sessions are necessary because we did not observe additional gains at the 1-month time point, following three weekly “booster” tACS sessions. Further research will be required to understand whether these weekly booster sessions had no effect, or whether they served to maintain the benefits achieved after the first week. Given that individuals who need cognitive remediation the most (e.g., clinical populations) are also the ones who benefit most from neuromodulation^44^, additional research can also investigate whether the number of tACS sessions may be contingent on the magnitude of cognitive decline (i.e., amount of room available for improvement).

Our previous research with theta tACS coupled with multitasking CCT found that individual differences in older adult neuroanatomy and neurophysiology both predicted tACS efficacy^38^. As this study was done entirely in the participant’s home, we did not collect neural data prior to tACS + CCT. Therefore, we were unable to individualize the tACS stimulation for each participant. However, we did apply a higher intensity (1.5 mA) compared to our prior research in healthy younger and older adults (1 mA) to account for potential cortical atrophy that would otherwise limit the amount of current that reaches the brain. Future research would benefit from collecting structural magnetic resonance imaging data to generate individualized models of electrical current flow that would allow for the optimization of current dose^45^. Similarly, greater tACS efficacy can be achieved by matching the stimulation frequency to the individual’s endogenous peak frequency evoked by the trained task^38,46^. Therefore, taken together with the established feasibility of conducting remote tACS + CCT, individually tailoring the dose and frequency of tACS protocols per individual should lead to even greater cognitive benefits^38^.

Finally, remote neurostimulation research has primarily been conducted in clinical populations, where researchers were often present via teleconferencing (reviewed in:^47,48^). Here, we take an important step and demonstrate that even an online researcher presence is not necessarily required for an md-aMCI population to improve aspects of cognitive control. However, we believe that this success was bolstered by in-person instruction prior to beginning the intervention, clear and concise written instructions to take home, and a calendar of events to help the participant track progress. Of course, as participants get closer to dementia, in-person assistance may become necessary. In this sample, our md-aMCI participants scored mostly at ceiling on the IADL and were able to navigate themselves to UCSF for consent and in-person instruction. Additional research in lower-functioning MCI or demented populations will be required to understand whether a critical point of effectiveness exists. This would inform feasibility of conducting at-home interventions across different clinical stages, and it would also provide important knowledge as to whether advanced disease progression may limit benefits of tACS + CCT. Understanding when (and whether) cognitive decline can be slowed or reversed prior to permanent loss of cognitive control is vital to overall public health.

## Methods

### Participants

**Figure 3:**
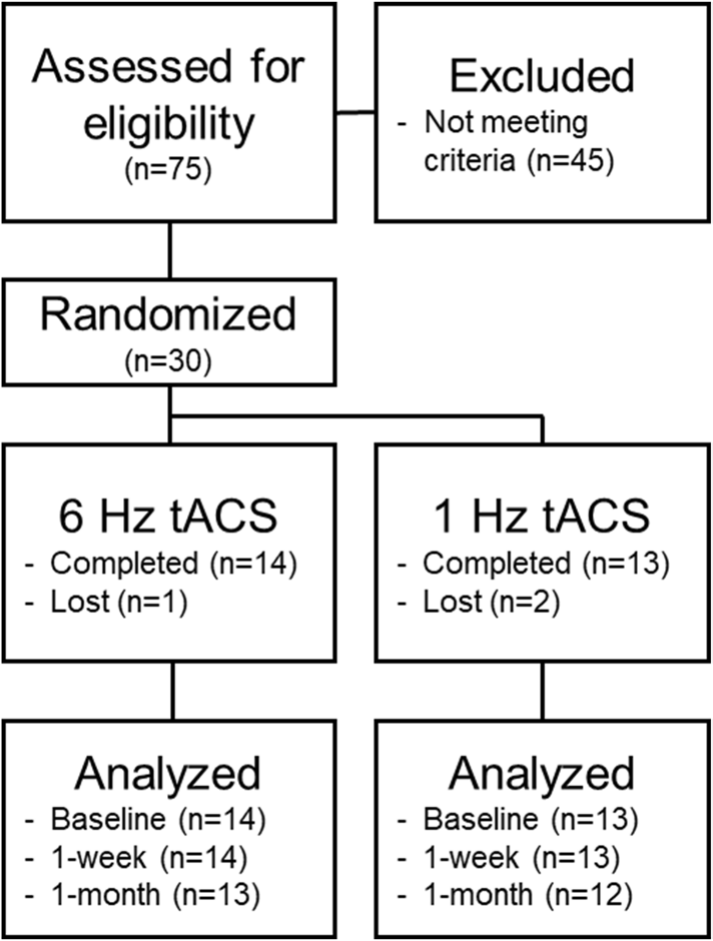
Participant eligibility, enrollment, group assignment, and analysis CONSORT flow chart.

In this double-blind randomized clinical trial parallel-group study, we enrolled participants aged 60–80 (Fig. 3). To be categorized as md-aMCI, participants scored between 17 and 28 on the Montreal Cognitive Assessment (MoCA^49^), had an age-matched Z score of at least − 1 on immediate memory or delayed memory (as measured by the California Verbal Learning Test^50^) *and* at least − 1 Z score on verbal and semantic fluency (D words, animals), processing speed (digit symbol and number trails tasks), or task switching (number letter trails task). Finally, participants needed a self-reported memory complaint.

Participants were randomly assigned following simple randomization procedures (computerized random numbers) to one of two treatment groups. The patients and experimenter assessing adherence were blinded to the random group assignment. We anticipated a 10% attrition rate, in line with our previous tACS studies^17,18,38,39^. To be included in this study, participants had to be English speaking, have at least 12 years of education, have normal or corrected to normal vision (without glaucoma, macular degeneration, amblyopia, or strabismus) and hearing, be able to complete cognitive tasks and study procedures, and be able to tolerate tACS.

Of the 30 participants, one dropped out for personal reasons and two participants did not adhere the CCT schedule during the initial week. This resulted in 27 participants who were randomly assigned to receive theta tACS (N: 14; Table 3) or control tACS (N:13; Table 3). All participants signed informed consent documents, which were approved by the University of San Francisco, California Institutional Review Board (IRB). All methods were carried out in accordance with relevant guidelines and regulations and were approved by the University of San Francisco, California IRB. Participants received $20 per hour for participation and a $50 bonus for completion of the study.

**Table 3:** Data are means (SD) or numbers (%).

	Theta tACS (N = 14)	Control tACS (N = 13)
Age (years)	70.4 (6.45)	69.5 (4.52)
Sex (female)	9 (64.29%)	4 (30.77%)
Handedness (right)	13 (92.86%)	12 (92.31%)
MoCA (score)	25.57 (3.48)	25.55 (1.86)

### Study procedures

**Figure 4:**
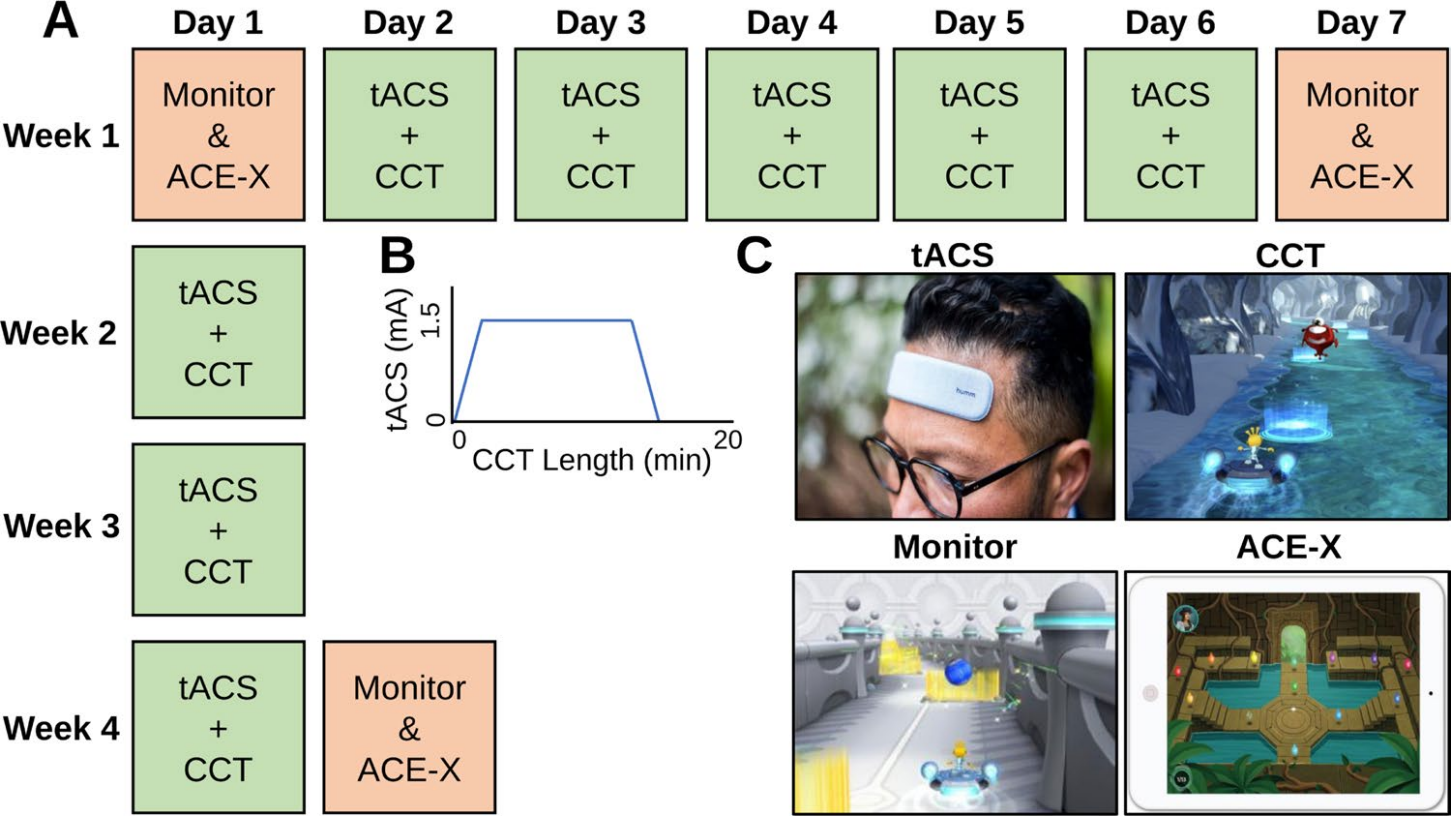
(**A**) Study timeline of events. The light orange boxes represent days where participants complete the outcome measures of Monitor, ACE-X, and the IADL survey. Light green boxes represent days where participants applied tACS while completing the CCT task AKL-T01. Each day in Week One occur consecutively without any gap days. (**B**) The strength of the tACS current during the length of the CCT task. The current ramped up and down to 1.5 mA over the course of 30 s at each end of the stimulation length. (**C**) Example image of the humm patch. Example screen shots of the CCT, Monitor, and ACE-X.

This study was preregistered at ClinicalTrials.gov with identifier NCT04647032 (first registered 11/30/2020, see Supplementary Information for procotol). In this study, participants first met with researchers at the University of California, San Francisco and provided informed consent. Participants received two iPad tablets, one for tACS days and one for outcome measures days. Participants were instructed on the use of each device (with a brief demonstration in front of a mirror with the tACS patches), the timeline of events, and provided with detailed written instructions. Once home and with active internet, participants first completed the outcome measure tasks without any neurostimulation (baseline). On the following day, participants self-applied the neurostimulation device and then completed 20-min of adaptive CCT. Participants completed the paired tACS + CCT task in the same manner for the following four days, totaling five consecutive days. On the following (sixth) day, participants completed the same outcome measures as baseline without any concurrent tACS (1-week assessment). The next day, one week from the initial session, participants completed the adaptive CCT task with paired tACS again. Participants then had two additional weekly sessions of tACS + CCT. Finally, on the day after the last (eighth) tACS session, participants completed a final tACS-free outcome measures session (1-month assessment; Fig. 4). In our past research employing tACS with paired cognitive training, we observed behavioral gains following just three sessions in healthy older adults^28,38^ and five in healthy younger adults^39^. Here, we assess gains in outcome measures at the 1-week and 1-month time points to measure gains and how additional sessions maintain or further rescue cognitive gains in MCI patients.

### Neuromodulation

Participants were randomized to receive theta tACS (6 Hz) or control stimulation (1 Hz) using a self-applied humm tACS device (humm, California, USA). Humm tACS devices consist of a single patch with two electrodes covered in adhesive gel that fixes directly below the hairline and extends from AF3 to AF4 (10–20 EEG system, Fig. 4C). During each CCT session, tACS was applied for 15 min at 1.5 mA (baseline-to-peak; 3 mA peak-to-peak). Stimulation also included 30 s of ramping up from 0 mA to full intensity and 30 s of ramping down to 0 mA at the end of the 15 min, for a total of 16 min (Fig. 4B). Participants first cleansed their forehead with an isopropanol wipe where the humm device was to be applied, then pressed a button at the center of the patch to begin the stimulation. After an automated impedance check, stimulation began and then automatically ended after the prescribed 16-min duration. Participants were provided with four humm patches as each patch was used twice across the eight stimulation sessions. Following the end of each tACS session, participants filled out a survey of side effects by rating the following 11 measures on a scale from 0 (not noticeable) to 10 (not tolerable): headache, neck pain, scalp pain, tingling, itching, burning sensation, increased alertness, increased sleepiness, trouble concentrating, acute mood change, phosphenes.

### Cognitive control training

On each of the eight CCT sessions participants began the self-applied tACS and immediately began the multitasking CCT task, *AKL-T01* (Akili Interactive Labs, Inc; Fig. 4C). AKL-T01 is a proprietary system based on patented technology underlying the NeuroRacer paradigm that challenges cognitive control by requiring multitasking performance (for more details see:^14^). Furthermore, AKL-T01 is significantly easier than NeuroRacer to perform for older adults, particularly those with MCI, given the constant difficulty adjustments and ease of use of a tablet. We previously employed AKL-T01 with at-home remote tACS in healthy younger adults to measure cognitive gains^39^. Briefly, participants guide a character down a path by tilting the iPad like a steering wheel (visuomotor/sensorimotor task). At the same time, participants were engaged in a perceptual discrimination task, where they tapped on the screen in response to target items (e.g., green fish) and ignored all distractors (e.g., blue fish). Importantly, AKL-T01 employs algorithms that continuously adapts to individual performance in real time with feedback provided. Correctly identifying consecutive targets lowered the response time to be counted as correct, visually depicted as faster moving targets. Consecutive obstacles (gates) avoided in the visuomotor task increased the speed of the vehicle and misses slowed down the speed. Participants completed five ‘missions’ per day, which lasted approximately 20-min in total (i.e., four minutes longer than tACS). Researchers were able to remotely monitor adherence to the CCT sessions as performance data was uploaded online during each session.

### Outcome measures

Primary outcome measures consisted of (1) multitasking ability on the CCT task, (2) a sustained attention task and (3) a working memory task. On each of the three sessions where outcome measures were assessed (baseline, 1-week, 1-month), participants completed the outcome assessments on a separate color-coded iPad than the CCT task. To assess multitasking ability on the CCT task, participants completed Monitor (AKL-M01, Akili Interactive Labs, Inc), which is an assessment version of AKL-T01. Monitor consists of a set of game-like tasks lasting approximately seven minutes in which participants were engaged in a dynamically adjusting perceptual discrimination task with and without concurrent visuospatial tracking^51^. Both discrimination and tracking tasks use adaptive algorithms that continuously adjust task difficulty to converge on a consistent proportional correct value, which is saved as an individual threshold of discrimination and tracking performance. These threshold values were subsequently used in statistical analyses to assess multitasking performance.

Participants also completed the Adaptive Cognitive Evaluation-Explorer (ACE-X)^52^. The tasks in ACE-X are standard tests that assess different aspects of cognitive control (attention, working memory), modified by incorporating adaptive algorithms, immersive graphics, video tutorials, motivating feedback, and a user-friendly interface. Each task within ACE-X was completed in approximately five minutes. Two of these tasks were sustained attention tasks used to assess attentional control, and were modeled after the Test of Variables of Attention task (TOVA^53^). In both tasks, participants were instructed to respond as quickly as possible to a target stimulus that appeared at the top of the screen and ignore any stimuli that appeared at the bottom of the screen. Both stimuli were presented for 100 ms, had 2.1 s to respond, and trials occurred every two seconds. The task with frequent stimuli (32 of 40 trials) assesses inhibitory control abilities. The task with infrequent stimuli (16 of 80 trials) assesses sustained attention abilities. There was no adaptivity of response window or feedback for these tasks to closely follow the design of the TOVA.

To assess working memory, two ACE-X tasks were used (based on the Corsi block task^54^), which measures visuospatial working memory capacity (WM). Briefly, a field of randomly distributed diamonds appeared on the screen and participants were to tap the order (or backwards order) of the diamonds that were illuminated for one second each. Participants begin with three targets and if they complete two consecutive trials successfully, they advance to the next level with one additional target. If a participant fails three trials in a row, the task ends. Participants can complete up to a total of nine targets. Participant must select all target gems in the correct order within the maximum response time (five seconds + one second per target).

The secondary outcome measures consisted of the Instrumental Activities of Daily Living (IADL)^55^ survey, where participants rated their level of independence (independent, needs help, dependent, or cannot do) on 16 metrics (e.g. driving, managing finances, bathing) on a scale of 1 (cannot do) to 4 (independent). Mean ratings across all metrics were used for statistical analysis of IADL.

### Exploratory outcome measures

In addition to our primary outcome measures assessing multitasking, sustained attention, and working memory, we also collected data from four other ACE-X tasks to be used in an exploratory analysis of other aspects of attentional control: (1) Boxed, a visual search interference processing based on the visual search paradigm^56^. (2) Color Tricker, based on the Stroop task^57,58^ measuring central visual inhibitory control. (3) Flanker, a peripheral visual inhibitory control task^59^. (4) Compass, a spatial selective attention task based on the Posner cueing paradigm^60^.

Boxed is a forced-voice task with four stimuli conditions of 20 trials each: set size of four or 12 and feature or congruent stimuli. The stimuli are colored landolt squares (red and green) with side openings (left, right, bottom, or top) and participants are instructed to attend to the green with top or bottom openings (target) and ignore all other red and green squares. Participants respond to whether the target has an opening on the top or bottom. The feature four condition includes one green target and three red distractors. Feature 12 includes 11 red distractors and one green target. Conjunction four includes the green target, green distractor, and two red distractors. Conjunction 12 includes the green target, six red distractors, and five green distractors.

Color tricker, based on the Stroop task^57,58^, is designed to measure response inhibition performance. Participants viewed colored words that spell a color and are instructed to identify the color of the word (target) and ignore the color the word spells (distractor). The task has two conditions, congruent, matching color and word, and incongruent, mismatching color and word. In both conditions participants selected the color that matches the color of the word regardless of what is spelled. Each condition has 20 pseudorandomized trials with an equal number of targets of each color. A target color was never the word from the previous trial and a target color was never repeated two trials in a row.

Flanker is designed to measure selective attention and interference resolution performance. Participants view an array of five arrows and are instructed to identify the direction of a central arrow (target) surrounded by four flanking arrows (distractors). In congruent trials (14 trials) the center arrow points the same direction as the four flanking arrows and in incongruent trials (14 trials) the target arrow points in the opposite direction as the four flanking arrows. Trial types are mixed pseudo randomly such that no more than three trials in a row are the same condition.

Compass is based on the Posner cueing paradigm^60^ and is designed to measure spatial selective attention. Participants were instructed to look at the center of the screen where they saw an arrow pointing to the likely location of a target symbol. The task has three conditions, 40 valid trials (arrow points in correct direction), 10 invalid trials (arrow points in incorrect direction), and 10 neutral trials (arrow points in both directions). In each condition the participant tapped the side of the screen where the symbol appeared regardless of the direction the arrow was pointing. All trials are mixed pseudo randomly with no more than three of the same targets in a row (e.g. left) and no more than two invalid trials or 10 valid trails in a row.

On all adaptive ACE-X tasks, the window to respond was adaptive based on accuracy from the previous trial. This allows for the same tasks to be used in a range of participants that vary in age and clinical condition. If the participant is correct and responds quickly enough, the response window decreases by 10 ms. If the participant responds incorrectly or too slow, the response window increases by 40 ms. The minimum response window floor is 150 ms. For all adaptive modules, the inter-trial interval was 800–1200 ms and feedback occurred for 200 ms post response.

### Analyses

To measure protocol adherence, we conducted independent-samples t-tests between the tACS groups on the percentage of tasks completed as compared to what was expected (CCT: eight AKL-T01 sessions, Outcomes: three Monitor sessions, three ACE-X sessions, three IADL surveys). Side effects were averaged across the eight CCT sessions per participant for each of the 11 categories. We then conducted independent-samples t-tests between the two tACS groups for each of the 11 categories.

To assess divided attention (multitasking), we measured the difficulty threshold level reached at each time point for both the perceptual discrimination and visuomotor tracking multitasking modules. To assess sustained attentional control, we averaged RT across both portions of the ACE-X continuous performance task (frequent and infrequent targets). To assess working memory, we averaged the maximum span correctly achieved on the forwards and backwards span tasks. A Greenhouse–Geisser correction was used when appropriate. Effect sizes in ANOVAs are reported as partial eta squared ($\upeta _{{\text{p}}}^{{2}}$) and with Cohen’s d for independent samples t-tests. Post-hoc comparisons within rm-ANOVAs were Bonferroni-corrected.

### Exploratory analyses

To assess inhibitory control on the Flanker and Color Tricker tasks, we measured the average rate correct score (RCS) together for all trial types, which was defined as the number of overall correct trials divided by mean response time. To assess attentional orienting on the Compass task, we analyzed RCS across all trial types together. To assess visuospatial search on the Boxed task, analyzed RCS across all trial types together. For all outcome measure comparisons, we conducted repeated-measures ANOVAs (rm-ANOVA) using JASP^61^, with a within-subjects factor of time (baseline, 1-week, 1-month) and a between-subjects factor of tACS group (theta or control).

## Data Availability

The datasets used and/or analyzed during the current study available from the corresponding author on reasonable request.
